# A High-Density Gene Map of Loblolly Pine (*Pinus taeda* L.) Based on Exome Sequence Capture Genotyping

**DOI:** 10.1534/g3.113.008714

**Published:** 2013-11-05

**Authors:** Leandro Gomide Neves, John M. Davis, William B. Barbazuk, Matias Kirst

**Affiliations:** * Graduate Program in Plant Molecular and Cellular Biology, University of Florida, Gainesville, Florida 32611; † School of Forest Resources and Conservation, University of Florida, Gainesville, Florida 32611; ‡ University of Florida Genetics Institute, University of Florida, Gainesville, Florida 32611; § Department of Biology, University of Florida, Gainesville, Florida 32611

**Keywords:** loblolly pine, exome sequencing, high-throughput genotyping, high-density genetic map, copy number variation

## Abstract

Loblolly pine (*Pinus taeda* L.) is an economically and ecologically important conifer for which a suite of genomic resources is being generated. Despite recent attempts to sequence the large genome of conifers, their assembly and the positioning of genes remains largely incomplete. The interspecific synteny in pines suggests that a gene-based map would be useful to support genome assemblies and analysis of conifers. To establish a reference gene-based genetic map, we performed exome sequencing of 14729 genes on a mapping population of 72 haploid samples, generating a resource of 7434 sequence variants segregating for 3787 genes. Most markers are single-nucleotide polymorphisms, although short insertions/deletions and multiple nucleotide polymorphisms also were used. Marker segregation in the population was used to generate a high-density, gene-based genetic map. A total of 2841 genes were mapped to pine’s 12 linkage groups with an average of one marker every 0.58 cM. Capture data were used to detect gene presence/absence variations and position 65 genes on the map. We compared the marker order of genes previously mapped in loblolly pine and found high agreement. We estimated that 4123 genes had enough sequencing depth for reliable detection of markers, suggesting a high marker conversation rate of 92% (3787/4123). This is possible because a significant portion of the gene is captured and sequenced, increasing the chances of identifying a polymorphic site for characterization and mapping. This sub-centiMorgan genetic map provides a valuable resource for gene positioning on chromosomes and guide for the assembly of a reference pine genome.

Loblolly pine (*Pinus taeda* L.) covers 11.7 million hectares of natural and planted forests in North America and provides 58% of timber in the United States and 16% in the world’s (Wear and Greis 2002). Loblolly pine is also an important species for comparative studies between gymnosperms and angiosperms, and genomic resources are becoming increasingly available to enable these studies (Mackay *et al.* 2012). For instance, single-nucleotide polymorphisms (SNPs) and microsatellites have been identified and applied to generate genetic maps (Elsik and Williams 2001; Eckert *et al.* 2009; Echt *et al.* 2011), identify population genetic parameters and associations to phenotype (Gonzalez-Martinez *et al.* 2007; Eckert *et al.* 2010; Stewart *et al.* 2012), and develop genomic selection prediction models (Resende *et al.* 2012). However, the number of available genetic markers remains small, particularly considering the large size of the loblolly pine genome.

Advances in high-throughput DNA sequencing and other genomic tools are making it possible to genotype large numbers of individuals by sequencing reduced representations of the genome (Davey *et al.* 2011). For example, targeted resequencing after *in solution* sequence capture (Gnirke *et al.* 2009) has proven useful in variant detection. Using this method, probes complementary to the target regions of the genome are designed and hybridized to genomic DNA for sequence capture and subsequent sequencing. Sequence capture is being optimized for an increasing number of plant species (Saintenac *et al.* 2011; Bundock *et al.* 2012; Zhou and Holliday 2012) including conifers (Neves *et al.* 2013). To evaluate the potential of targeted resequencing for genotyping in loblolly pine, we used sequence capture to detect polymorphisms based on the segregation of alleles in a recombining population.

The analysis of a recombining population allows for the construction of genetic maps in which markers are grouped and ordered on the basis of observed recombination frequencies. When the markers are located within genic regions, the genetic map provides the relative position of genes in chromosomes (Drost *et al.* 2009; Neves *et al.* 2011), which is particularly relevant for loblolly pine or other species without a reference sequence. High-density, gene-rich genetic maps are useful to examine genome synteny with other species (Krutovsky *et al.* 2004), for detection of quantitative trait loci and identification of candidate regulatory genes (Bernardo 2008), and to support genome assembly (Tuskan *et al.* 2006; Sato *et al.* 2012). Currently, the best-resolved loblolly pine genetic map was reported by Eckert *et al.* (2010), which positioned 1635 genes, genotyped from a starting SNP panel of 7535 markers. More recently, Echt *et al.* (2011) reported the identification of large numbers of microsatellites and their use to create a moderately dense map with 429 markers. A subset of 311 of these microsatellites was derived from cDNA and, therefore, reflects the position of the corresponding genes.

The goal of this work was to genotype a loblolly pine mapping population by the use of sequence capture to analyze the efficiency of this method for polymorphism detection and to create a high-density, gene-rich genetic map. The mapping population consisted of 72 haploid megagametophytes extracted from seeds of a single tree. In pine seeds, the haploid DNA from individual megagametophyte represents the product of recombination of the maternal genomic DNA. Sequence capture was performed in each haploid sample and 7842 markers were detected that segregate in the population, representing 4195 genes. The best marker for each gene was selected and 2843 genes were ordered in a genetic map, with an average of one marker every 0.58 cM. Sequencing depth data were used to detect 408 genes segregating for presence/absence variation of which 65 were ultimately mapped in the genetic map. The comparison of our genetic map with the previous map of Eckert *et al.* (2010) via 397 common genes showed high levels of agreement at the grouping and ordering level between the two maps.

## Materials and Methods

### Mapping population, DNA extraction, and sequence capture

Seventy-two seeds were collected from the reference loblolly pine genotype 17 (Kayihan *et al.* 2005). Each seed was manually dissected to extract the haploid, maternally inherited megagametophyte tissue. Megagametophytes were lyophilized and DNA extracted using the DNeasy Plant Mini Kit (QIAGEN) according to the manufacturer’s protocol. DNA sample quality and integrity was analyzed by agarose gel electrophoresis, and DNA samples were quantified using the Quant-it PicoGreen dsDNA Assay Kit (Invitrogen). Libraries for each haploid sample were created as described previously (Neves *et al.* 2013). In summary, DNA was sheared, end-repaired, adenylated, and ligated to Illumina compatible adapters that contain 5′ barcode sequences. Fragments with ligated adapters were size-selected (250−500 bp) by agarose gel electrophoresis and enriched by polymerase chain reaction using universal primers. Libraries from eight barcoded haploid samples were combined in equimolar amounts and used as input for the sequence capture hybridization reactions following the Agilent SureSelect protocol. The probe set used for sequence capture contained 54773 probes representing 14729 *P. taeda* unigenes and are available in a previous study (Neves *et al.* 2013). Target enriched libraries were sequenced using a variety of Illumina machines and outputs (Supporting Information, Table S1).

### Sequencing data filtering and alignment

Raw reads for each sequencing lane were separated on the basis of their individual barcode using the FASTX-Toolkit barcode splitter (http://hannonlab.cshl.edu/fastx_toolkit/), resulting in one file for each sample. The 3′ ends of the reads were trimmed to remove low-quality bases with the FASTX-Toolkit fastq quality trimmer with parameters -t 20 -l 50 (remove 3′ end bases if phred quality score < 20 and discard read if resulting read is shorter than 50 bp) for reads sequenced for 100 bp or longer, and -t 20 -l 30 for reads sequenced for 40 bp. For samples sequenced in paired-end mode, the order of the reads in each mate file was resynchronized with the use of custom scripts, and reads without an appropriate mate were treated as single-end. The filtered reads were aligned to the unigene reference used for probe design with Mosaik Aligner (http://bioinformatics.bc.edu/marthlab/) with parameters as follows: maximum percentage of mismatches 0.05 (-mmp 0.05), unique alignment mode turned on (-m unique), most accurate hashing strategy (-a all), and hash size 15 (-hs 15). Multiple alignments for the same sample were combined by the use of BamTools (Barnett *et al.* 2011) into a single alignment.

### Detection of SNP and PAV

SNPs, short insertions and deletions (indels), and multinucleotide polymorphisms (MNPs) were detected in a combined population analysis that use all individuals of the mapping population simultaneously with Freebayes v0.8.7 (http://bioinformatics.bc.edu/marthlab/) with parameters as follows: SNP probability 0.75 (–pvar 0.75), pairwise nucleotide diversity 0.01 (–theta 0.01), haploid mode (–ploidy 1), and accepting indels (–indels); MNPs (–mnps), minimum alternative allele frequency of 0.8 (–min-alternate-fraction 0.8) and minimum alternative allele count of 1 (–min-alternate-count 1). For a polymorphism to be considered for mapping, reference and alternative alleles had to be observed cumulatively in 50 or more individuals of the population, and the alleles had to segregate 1:1 in the population (χ^2^-test *P*-value 0.05). If more than two alleles were observed at a polymorphic position, a problem that might arise as the result of contamination with diploid tissue (*e.g.*, embryo), paralog capture, and sequencing error, the individuals with the two most frequent alleles in the population were considered in further analysis and the remaining were categorized as missing data. To detect presence/absence variants (PAV), a sequencing depth matrix was calculated with genes as rows, haploid samples as columns, and every observation being the average number of times each position of the gene was sequenced. The observations were coded as 1, when at least one read aligned to the gene region, or 0 when no read aligning to the gene region was detected. A test for 1:1 segregation for the presence of the allele was performed (χ^2^-test *P*-value 0.05) and PAV markers passing these criteria were used for mapping.

### Genetic map construction

The SNP, short indel, MNP, and PAV markers that exhibited segregation in the population were combined, and in case of gene redundancy, the marker with the least amount of missing data was used as a representative of the gene. The genetic map was constructed with JoinMap 3.0 (Van Ooijen and Voorrips 2001) with parameters as follows: population type haploid, grouping logarithm of odds (LOD) score ≥6, recombination fraction ≤0.4, ripple value = 1, using the Kosambi mapping function. JoinMap 3.0 builds a genetic map adding markers in three successive steps. The first and second steps remove markers that reduce the map goodness-of-fit or creates negative distances. The third step then adds all markers, ignoring the thresholds of goodness-of-fit and negative distances. The complete map was generated by the use of all three rounds to order all linked markers to the map. For a conservative map, we stopped the mapping process after round two to provide a better estimation of gene order, as this removes markers that contribute to unstable ordering. The group number (1−12) and the relative orientation of the markers within the group were defined following the genetic map published by Eckert *et al.* (2010) to facilitate comparisons between studies. Genome length and coverage were estimated following Echt *et al.* (2011). The genome length was estimated using method 4 of Chakravarti *et al.* (1991), where the observed Kosambi genetic distance of each linkage group (LG) was adjusted by multiplying it by (*m*+1)/(*m*-1), where *m* is the number of markers mapped to the LG. Genome coverage (*c*), defined as the proportion of the genome contained within a distance *d* (cM) from a marker, was estimated by $c=\;1-\;e^{-2dm/L}$, where, *L* is the total estimated genome length (Lange and Boehnke 1982; Remington *et al.* 1999; Echt *et al.* 2011).

## Results

### Genotyping of a mapping population with sequence capture

We used a multiplexed sequence capture method (Neves *et al.* 2013) to characterize genetic variants in 14729 genes. Seventy-two seeds were collected from a single female tree, and the maternally inherited, haploid megagametophyte tissue was used for library preparation. The use of DNA from the seed megagametophyte provides an effective way to assess recombination frequency between genetic markers, without the need for crosses (Adams and Joly 1980). The 72 haploid samples were captured in nine pools of eight megagametophytes each, and sequenced with a median depth of 2.8× across 54773 probes (average of 3.7 probes/genes). Of the 14729 genes targeted, 6283 (43%) were not captured and/or sequenced in any sample. This occurred either because these probes failed to hybridize to genomic DNA or because the captured fragments were not sampled in the sequencing process due to the shallow sequencing depth used. Probes were designed primarily on the basis of a transcriptome assembly, and in a previous analysis of probe performance (Neves *et al.* 2013) we discovered that a significant number of them were complementary to the boundary of two exons, justifying the high failure rate and illustrating a limitation of designing probes for species with uncharacterized genomes. For the remainder 8446 genes (57%), sequence capture was successful for at least one probe, for at least one individual. Of these, 4123 (28%) were sequenced in 50 or more haploid samples of the population. If sequencing data complementary to a given probe was available for fewer than 50 samples, the locus was excluded from further analysis. Therefore, from the 14729 genes initially considered for capture, 28% (4123) were actually captured in enough individuals, and sufficient sequencing depth was generated to allow identification of segregating sequence variants.

The analysis of the sequence data identified 7434 markers segregating 1:1 in at least 50 haploid samples of the population. These markers represented 3787 genes (Table 1). The majority of these markers were SNPs (6857), whereas short deletions, short insertions, and MNPs accounted for 210, 208, and 159 markers, respectively. The average sequencing depth for each gene was used to identify possible PAVs (Swanson-Wagner *et al.* 2010). The average sequencing depth at the gene level was used to compensate for variations that exist at the probe level and 408 putative PAVs segregating 1:1 in the mapping population were considered for mapping (Table 1). Altogether, 7842 segregating markers were identified for 4195 genes, with an average of 2 markers per gene and a range of 1 to 13 (Table 1). A list of the markers and their allele representation within the population is described in Table S2. Because the presence of multiple markers per gene provided redundant data for mapping, the marker with the least missing data were selected for the gene, resulting in a final set of 4195 markers for mapping.

**Table 1:** Description of markers segregating and mapped in the population

Marker Class	Segregating 1:1		Genes Mapped
	No. Marker	No. Genes	
Sequence variation	7434	3787	2776
SNP	6857	−	2622
Short deletions	210	−	63
Short insertions	208	−	53
MNP	159	−	38
Presence/absence variation	408	408	65
Total	7842	4195	2841

Markers identified based on sequence variation were subdivided in single-nucleotide polymorphism (SNP), short insertions and deletions (short indels) and multiple-nucleotide polymorphism (MNP). No. Markers represent the number of markers that segregated 1:1 in the mapping population, and No. Genes is the number of unique genes represented by these markers

### A *P. taeda* genetic map with 2841 gene markers

**Figure 1:**
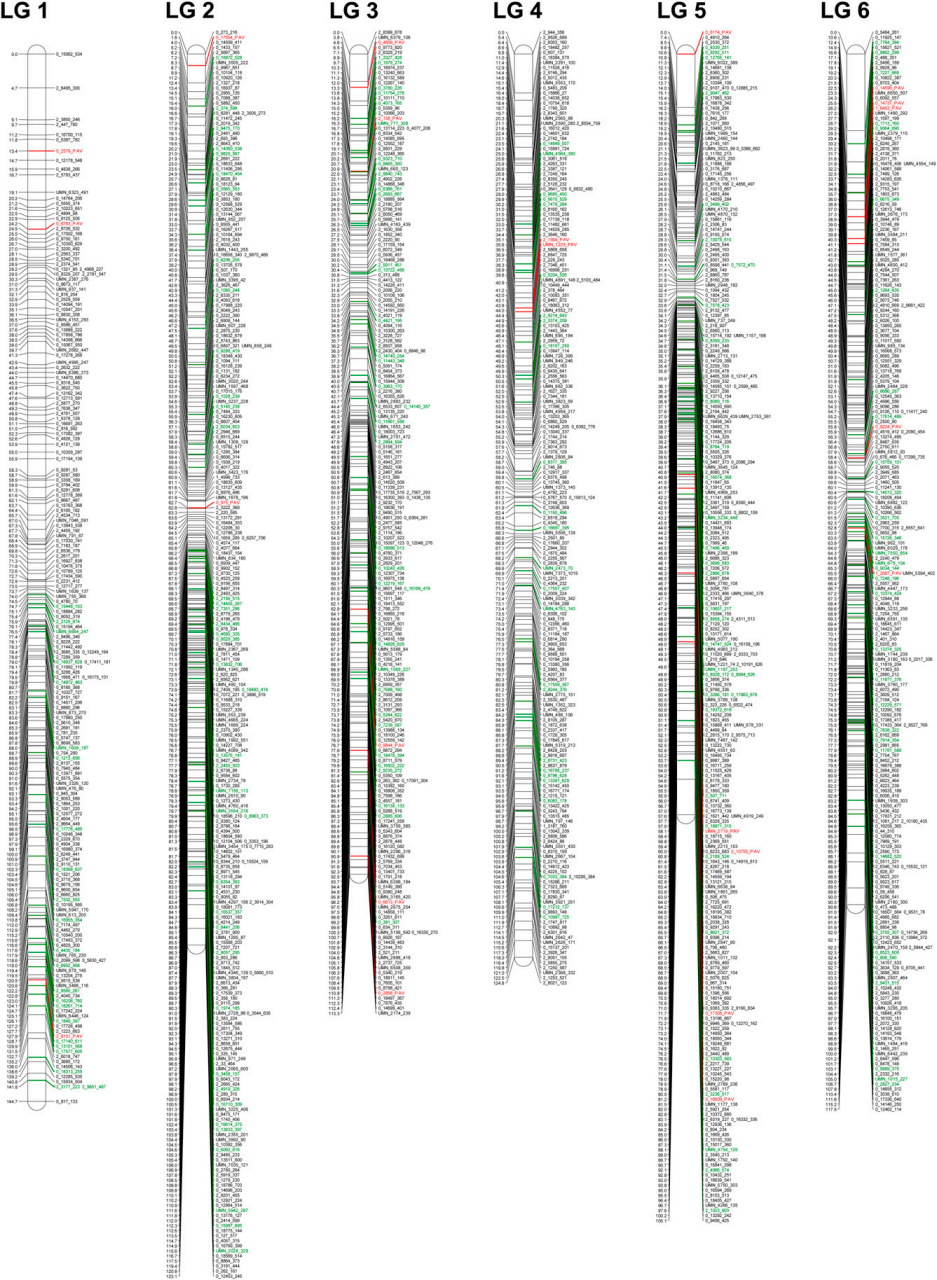

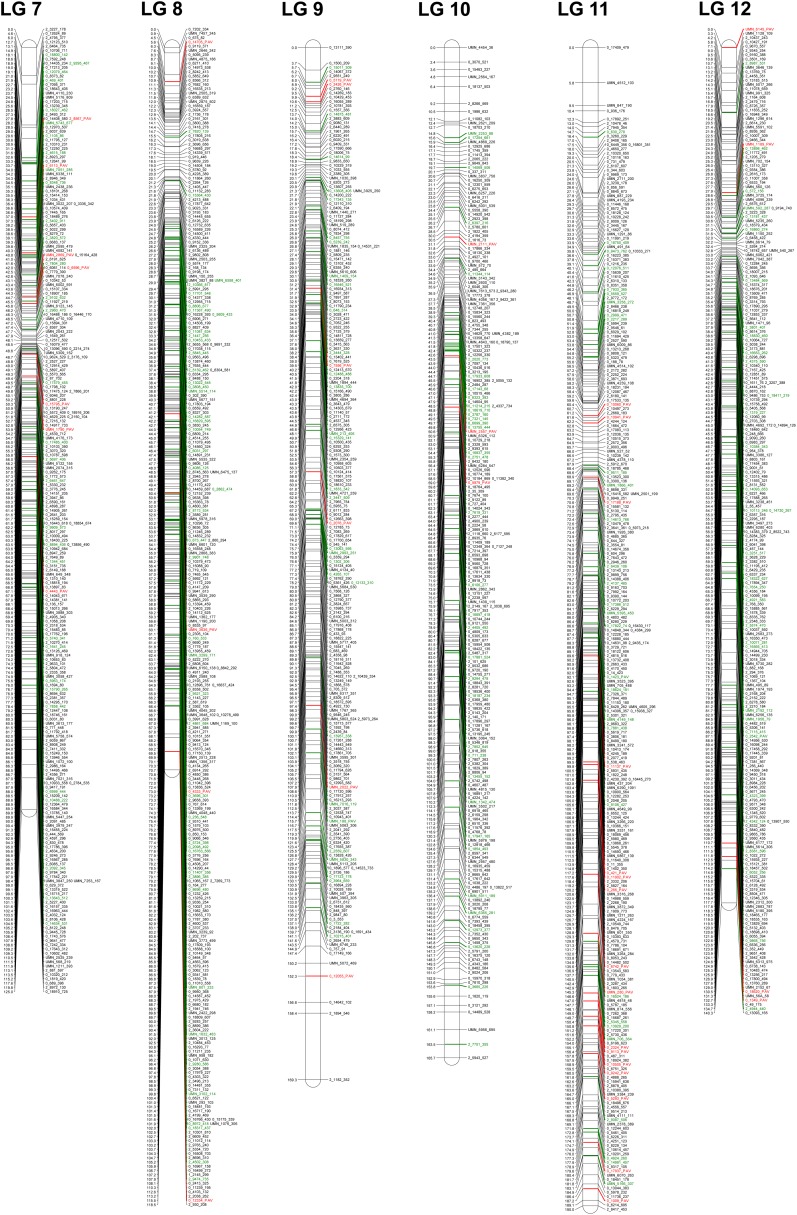
High-density genetic map of loblolly pine. The final map is composed of 2841 genes distributed among the 12 LGs with an average distribution of a gene every 0.6 cM and a total length of 1637.4 cM. For easy comparison, the LG name and orientation of the map follow that published by Eckert *et al.* (2009), and common genes between the two maps are shown in green. Genes for which presence/absence variation was identified and used to map the gene is shown in green and have suffix PAV (*e.g.*, 2_6131_PAV, for unigene 2_6131). The remaining markers were coded as “unigene_marker position” (*e.g.*, 0_2563_337, for unigene 0_2563 and a polymorphic marker at position 337 of the unigene). The genetic map graphical visualization was prepared using MapChart 2.2 (Voorrips 2002).

The segregation of 4195 markers in 72 haploid samples was used to construct a high-density, gene-based genetic map (Figure 1 and Table S3). The final map comprises 2841 genes mapped to the expected 12 LGs of the species. The number of genes mapped in each LG varied from 193 genes for LG 1, to 291 genes in LG 8, with an average of 237 genes per LG. Of the 2841 markers mapped, 2622 (92%) were SNPs, 63 (2%) were short deletions, 53 (2%) were short deletions, 38 (1%) were multiple nucleotide polymorphisms, and 65 (2%) were gene PAVs (Table 1). The final map spans 1637.4 cM, which is comparable with previous estimations of map length for the species (Remington *et al.* 1999; Eckert *et al.* 2010; Echt *et al.* 2011). LG 11 spanned the longest genetic distance with 190 cM, whereas LG 5 spanned the shortest, with 105.1 cM. On average, LGs spanned 132.9 cM (Table 2). The average interval between markers was 0.58 cM, across 12 LGs, with 95% of the intervals smaller than 1.5 cM, and a maximum interval of 10.9 cM at the end of LG 2. Following previous methods (Remington *et al.* 1999; Echt *et al.* 2011) we estimated the genome length at 1651.5 cM, and the map coverage as 99%. For this marker density, the estimated map coverage (c) predicts that any locus on the genome has 99% probability of being within 3 cM of a marker and 82% probability of being within 1 cM of a marker. A map with a more conservative marker order, obtained by stopping the mapping process at the second mapping step of JoinMap, was generated and is provided in Table S4. When considering this map, there are still 1371 genes mapped with an average of one marker every 1.3 cM, providing an estimated map coverage (c) that predicts that any locus on the genome has 98% probability of being within 5 cM of a marker.

**Table 2:** Description of genetic map

Linkage Group	No. Genes Mapped	Length, cM	Intermarker Distance, cM	
			Mean	SD
1	193	144.72	0.75	0.65
2	260	123.12	0.48	0.39
3	205	113.29	0.56	0.44
4	195	124.79	0.64	0.54
5	263	105.07	0.4	0.62
6	230	117.54	0.51	0.83
7	242	125.03	0.52	0.71
8	291	118.53	0.41	0.41
9	223	169.32	0.76	0.91
10	228	165.71	0.73	0.58
11	271	190	0.7	0.62
12	240	140.29	0.59	0.59
Average	236.75	136.45	0.58	0.61
Total	2841	1637.41	−	−

A total of 2841 genes were mapped to the expected 12 linkage groups of loblolly pine based on recombination data from 72 haploid samples.

### Map validation

The high-density map developed provides an overview of the grouping and orientation of 2841 genes in each loblolly pine chromosome. However, because the number of markers far exceeds the number of meiotic recombinations sampled to generate the map, it can be expected that their order might present incongruences with the physical position on the genome, particularly between closely mapped markers. To analyze the consistency of our map, we compared it with the genetic map built by Eckert *et al.* (2010), hereafter referred to as “*Eckert map*” because a large number of genes were shared between the two. Ideally, this comparison should be conducted relative to the genome sequence of loblolly pine, but the current assembly (version 0.8) is unordered and still largely fragmented. Thus, the comparison relative to another gene-based genetic map remains the only viable option to validate our results. The *Eckert map* was generated with SNPs detected by the use of an Illumina Infinium genotyping assay with markers selected from a unigene dataset that overlapped with the one used in our sequence capture probe design. Although the SNPs analyzed within the genes were not the same, a total of 397 genes had markers in common between the two maps and could be used for a comparative analysis. The analysis occurred in two steps: (1) First, we analyzed whether the common genes mapped to the same LG. (2) Next, we evaluated if the order of the genes within the LG were similar between the two maps.

**Figure 2:**
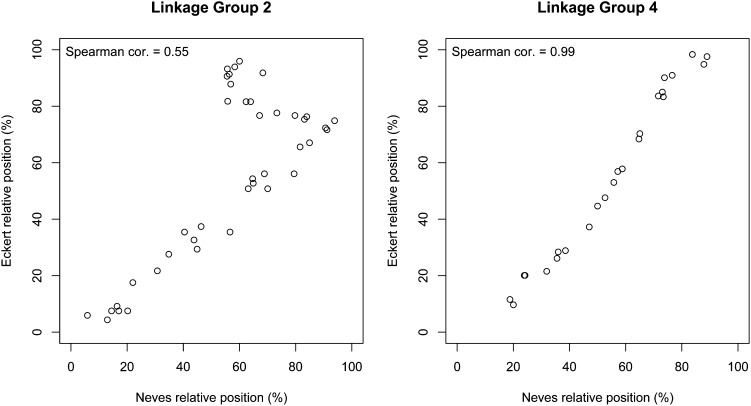
Comparison of the normalized relative order of shared genes used in our study (X-axis) and that of Eckert *et al.* (2009) (Y-axis). Because of space constraints, only plots for LGs 2 (left) and 4 (right) are shown. Assuming genes syntheny between the two populations, a straight line would illustrate perfect agreement at the gene ordering level between the two maps. Graphs for all 12 LGs are provided in Figure S1 and Figure S2.

In the first analysis, of the 397 genes positioned in both maps, 392 (99%) mapped to the same LG as that observed in the *Eckert map*. Thus, there is a nearly perfect agreement between the two maps at the grouping level (Table 3). For the comparison of the genetic position of genes within LGs, the data are illustrated by plotting the normalized position of the common genes on our map (X) relative to the normalized position on the *Eckert map* (Y) (for instance, LG 2 and LG 4 in Figure 2). In the comparative analysis, the position of each gene was normalized to control for the different length of the two maps. For example, a gene mapped to position 61.56 cM on LG 2, which has a length of 123.12 on our map (Table 2), would have a normalized position of 50%. The same approach was used for genes in the *Eckert map*. Assuming gene synteny between the populations used for mapping, the expected results would be a linear relationship between the markers. For LG 1, 3, 4, 8, 9, 10, and 11, all the common genes were collinear across the entire extension of the group (Figure 2, Figure S1, and Figure S2). For LG 2, 6, 7, and 12, most of the genes were collinear, but a block defined by several genes shows incongruences between the two maps (Figure 2, Figure S1, and Figure S2). Interestingly, in all cases, these blocks were previously mapped to the beginning or end of the LG in the *Eckert map* and were mapped toward the middle of the LG in our map. In only one LG, LG5, there is low agreement between the two maps (Figure S1, and Figure S2). Overall, the results show a high agreement between the genes mapped in the two maps, both at the grouping level and at the ordering level.

**Table 3:** Marker validation at the grouping level: comparison of the maps from Eckert *et al.* (2010) and the present map

LG	No. Genes at Same Group	No. Genes at Different Group	Genes Linked to Different Groups
1	23	0	
2	40	0	
3	35	0	
4	24	1	Gene = 0_9680; Neves = LG 4, Eckert = LG 8
5	33	1	Gene = 0_7496; Neves = LG 5, Eckert = LG 11
6	32	0	
7	31	1	Gene = 0_5740; Neves = LG 7, Eckert = LG 12
8	45	0	
9	30	1	Gene = UMN_213; Neves = LG 9, Eckert = LG 7
10	35	1	Gene = 0_6106; Neves = LG 10, Eckert = LG 6
11	30	0	
12	34	0	
Total	392	5	

The few genes mapped to different linkage groups (LG) between the two studies are provided in the last column.

## Discussion

The two main objectives of this study were to evaluate the potential of targeted resequencing on the basis of sequence capture for polymorphism detection in loblolly pine and to expand our knowledge about the position of genes in the pine genome. Loblolly pine is one of the most economically and ecologically important species within the conifers, and its genome is currently being sequenced (http://pinegenome.org/pinerefseq). The large and complex pine genome, characterized by large retrotransposons expansions and high nucleotide diversity (Brown *et al.* 2004; Burleigh *et al.* 2012), offers challenges for polymorphism genotyping when traditional platforms, such as SNP chips, are used, resulting in low rates of SNP conversion and high rates of missing data (Eckert *et al.* 2009). From previous work (Neves *et al.* 2013) we expected that probes used for sequence capture could be capturing nonunique regions in the pine genome (*e.g.*, paralogs and pseudogenes), hampering the detection of site-specific polymorphisms. The use of a mapping population provides a method to verify detected polymorphisms because alternative alleles are expected to segregate in equal proportion (1:1) in a sample of megagametophytes. Even when adopting a shallow sequencing depth, we detected a total of 7434 sequence variant markers for 3787 genes (Table 1) segregating in the population. From the 14729 genes initially considered for capture, we estimated that 4123 (28%) had sequence properties that allowed us to detect sequence variation, namely having at least 50 individuals with one or more reads aligned to the gene in each individual. Thus, given appropriate sequence capture efficiency and sequencing depth, 92% (3787/4123) of the genes contained at least one segregating marker that was used for mapping in the population. This is a high SNP conversion rate considering that no previous information was available for the population. A greater number of genes likely could have been mapped if we had sequenced the population at higher depth or increased the number of megagametophytes sequenced to sample more meiotic events.

The megagametophyte used as source of DNA sequence capture is a haploid, maternally inherited tissue that is commonly used in conifer mapping studies (Remington *et al.* 1999). Thus, a single haplotype was anticipated for any given locus. The pipeline for detection of sequence polymorphism required that the most common base detected in a given megagametophyte be present in at least 80% of the reads that overlap the variant locus. In other words, for a particular position with sequencing depth of 10×, eight or more reads need to have the same base to be considered. This step was implemented to exclude possible errors added during library construction and sequencing, and to avoid cases where unspecific paralogs and pseudogenes were also being captured, which is likely considering their abundance in the pine genome (Kovach *et al.* 2010). Although we did not formally quantify instances where the latter happens, we observed it while optimizing the bioinformatics pipeline. For example, only reads aligned to the unigene reference with less than 5% mismatch were considered for polymorphism detection and mapping. At this threshold, we detected the 7434 sequence polymorphisms reported. When up to 10% mismatch to the unigene reference were permitted for use of a sequencing read, there was a significant reduction in the number of polymorphisms detected that segregated in the expected ratio in the population. Accepting 10% of mismatches lead to the detection of only 4563 polymorphisms, represented within 2210 genes, that segregate in a 1:1 ratio. This finding suggests that *bona fide* polymorphisms were confounded by alleles from paralogs and pseudogenes mapped with less stringent alignment criteria.

The large size of the pine genome of pine is attributed to retrotransposon activity, rather than whole-genome duplications (Mackay *et al.* 2012). On the basis of the dynamic nature of retrotransposons, we hypothesized that a subset of the pine genes might be present in multiple copies in the genome, as observed in maize and another genomes characterized by retrotransposon genome expansions (Belo *et al.* 2010; Swanson-Wagner *et al.* 2010). To test this hypothesis, we aimed to detect PAVs. In the specific case of a maternally derived haploid segregating population, these are hemizygous genes that segregate in the population with 50% of the haploid samples containing the gene in their genome, and 50% with the gene absent in their genome. We focused on PAVs, because these events are easier to detect than copy number variations (Teo *et al.* 2012), particularly considering that samples were sequenced at low depth. In our analysis, a gene was considered absent when no reads aligned throughout the entire gene region, and present when at least one read was aligned to the gene region. Using this simple analysis criteria allowed us to detect 408 putative PAV. However, only 16% (65) of the PAVs could be mapped, suggesting that many of PAVs were false positives or contained genotyping errors that excluded them from mapping. These false positive or genotyping errors were expected as a consequence of the low sequencing depth, and we anticipated that the genetic mapping process would be able to filter those out, providing a more accurate subset of PAVs in *P. taeda*.

The final genetic map was obtained by analyzing the segregation of 4195 markers, each representing a unique gene of the *P. taeda* genome, in 72 haploid samples. Ultimately, 2841 (68%) genes were mapped in the expected 12 LGs. To the best of our knowledge, this is the most dense genetic map for conifers or trees in general, although other highly saturated maps are becoming available (Martinez-Garcia *et al.* 2013). Because this map was constructed using gene-based marker, it provides links with other published maps for loblolly pine, allowing map cross-checking and eventual consolidation. The genes mapped were not selected based on any specific criteria. Thus, the map provides an unbiased representation of the loblolly pine gene set that can be used for studies that benefit from a preliminary ordering of the genes until a reference genome sequence of reasonable quality becomes available. This high-density map should also be valuable when anchoring and orienting scaffolds in the assembly of the reference genome (Tuskan *et al.* 2006; Sato *et al.* 2012).

The comparison to a previous map of *P. taeda* (Eckert *et al.* 2010) that shares 397 common genes with our new map revealed a high-level of agreement at the grouping and ordering level (Table 3). These results provide strong evidence that markers were correctly identified and genotyped in the mapping population, despite the complexity of the genome of pine. For some LGs (2, 6, 7, 8, and 12; Figure 1, Figure S1, and Figure S2), blocks of adjacent markers that were previously assigned to the edge of the LG in the map of Eckert *et al.* were mapped toward the center of the LG in our map. Such discrepancies between blocks of genetic markers have been observed in other studies in *Pinaceae* (Krutovsky *et al.* 2004; Eckert *et al.* 2009; Echt *et al.* 2011) and were usually attributed to genotyping errors. Although we cannot test for genotyping errors, we did not observe higher rates of missing data in the markers that are out of order compared to the collinear ones (data not shown). Also, from simulated experiments, it is expected that genotyping errors would inflate the length of the genetic map (Hackett and Broadfoot 2003), but this was not observed in our map. Therefore, we are unable to conclusively identify the causes for noncollinear blocks of markers. Attributing such discrepancies to real genome rearrangements will require further experiments based on larger numbers of individuals or whole-genome resequencing.

Genotyping samples of a large population using sequence capture presents some advantages over conventional genotyping methods. For example, sequence capture does not require one to previously characterize the polymorphisms to be detected. We show that this is of greater importance for a segregating population because even if previously characterized polymorphisms are available, it is not known which of those sites will segregate in a single biparental segregating population and have the potential to be mapped. In this study, the probes used for capture were designed to span the entire unigene length, and we were able to detect an average of two polymorphic sites segregating in the population for every gene. Thus, the chances of detecting a marker to map the gene increased and we were able to map 68% of the genes containing a segregating marker (2841/4195), even adopting an overall shallow sequencing depth of 2.8×. For several genes we detected additional polymorphic sites within 60 bp of the position used for mapping, without impairing the efficiency of the capture. This is an usual observation in pine and other highly polymorphic species, and a common limitation for developing traditional genotyping methods, such as SNP chips (Eckert *et al.* 2009; Grattapaglia *et al.* 2011). Finally, another advantage illustrated is the potential to detect other types of genetic variants in addition to SNPs, such as the presence/absence markers that we were able to detect and map for 65 genes. Although a small percentage of the mapped markers were genotyped with PAV, sequencing at higher depth will likely increase this number and enable more accurate detection of gene copy number variation, as achieved by other studies in plants (Saintenac *et al.* 2011; Valdes-Mas *et al.* 2012). With an ultimate goal to use genetic markers for phenotypic characterization, recent studies are showing that copy number variations might explain a portion of phenotypic variation not sampled by SNPs, justifying its characterization (Maron *et al.* 2013).

We report the application of exome capture on loblolly pine for polymorphism detection and gene mapping by analyzing a mapping population of 72 haploid samples. An important advantage of this approach for polymorphism detection is that it allows for the enrichment and sequencing of several segments of the gene being targeted, which increases the chances of detecting a marker segregating in the population. As a result, we were able to detect at least one segregating marker for 4195 genes, despite the shallow sequencing depth employed. The markers were used to generate a gene-rich genetic map that ultimately positions 2841 genes with an average intergenic distance of 0.6 cM. Also, because exome capture is a genotyping by sequencing method, we show that other types of segregating markers can be identified in the population, such as gene presence/absence variation. This is particularly important, because it highlights the possibility to study gene dosage using exome capture in plants, which traditionally has required additional experimentation. To our knowledge, this is the most saturated map in conifers and trees in general. Because this is a gene-based map, we expect that it will help in the construction of the pine reference sequence that is currently being generated while facilitating other basic and applied research efforts that use a high-density, gene-rich genetic maps.

## Supplementary Material

Supporting Information
