# Supplementary material for: A High-Density Gene Map of Loblolly Pine (Pinus taeda L.) Based on Exome Sequence Capture Genotyping
Source: G3 (Bethesda). 2013 Nov 5;4(1):29–37. doi: 10.1534/g3.113.008714 (PMC3887537; doi:10.1534/g3.113.008714)
Supplement: Supporting Information [file supp_4_1_29__index.html]

A High-Density Gene Map of Loblolly Pine (Pinus taeda L.) Based on Exome Sequence Capture Genotyping — A High-Density Gene Map of Loblolly Pine (Pinus taeda L.) Based on Exome Sequence Capture Genotyping — Supporting Information 

# A High-Density Gene Map of Loblolly Pine (*Pinus taeda* L.) Based on Exome Sequence Capture Genotyping

## Supporting Information for Neves *et al.*, 2014

**Files in this Data Supplement:**

- Supporting Information - Figures S1-S2 and Tables S1-S4 (PDF, 626 KB)
- Figure S1 - Comparison of the normalized relative order of shared genes used in our study (X-axis) and that of Eckert *et al.* (2009) (Y-axis) for linkage groups one to six. (PDF, 362 KB)
- Figure S2 - Comparison of the normalized relative order of shared genes used in our study (X-axis) and that of Eckert *et al.* (2009) (Y-axis) for linkage groups seven to twelve. (PDF, 364 KB)
- Table S1 - Sequencing strategy used for each multiplexed pool of eight haploid samples. (PDF, 398 KB)
- Table S2 - Additional information about the 7,842 markers that segregated in the population (.csv, 504 KB)
- Table S3 - Text representation of the full genetic map with 2,841 genes mapped, containing the linkage group, the marker name and the position that marker mapped in the linkage group (.csv, 57 KB)
- Table S4 - Text representation of the genetic map of 1,371 genes mapped using the more conservative marker ordering of JoinMap Round 2 (.csv, 27 KB)
