# Supplementary material for: A High-Density Gene Map of Loblolly Pine (Pinus taeda L.) Based on Exome Sequence Capture Genotyping
Source: G3 (Bethesda). 2013 Nov 5;4(1):29–37. doi: 10.1534/g3.113.008714 (PMC3887537; doi:10.1534/g3.113.008714)
Supplement: Supporting Information [file supp_g3.113.008714_008714SI.pdf]

G3 · Genes | Genomes | Genetics

A high-density gene map of loblolly pine (*Pinus taeda* L.) based on exome sequence capture genotyping

Leandro Gomide Neves[1], John M. Davis[1,2,3], William B. Barbazuk[1,3,4§], Matias Kirst[1,2,4§]

[1]Graduate Program in Plant Molecular and Cellular Biology; [2]School of Forest Resources and Conservation; [3]University of Florida Genetics Institute; [4]Department of Biology, University of Florida, Gainesville, Florida, 32611.

[§]Corresponding author

Corresponding authors:
William B. Barbazuk:
2033 Mowry Road, room 407
Gainesville, FL, 32610
+1 (352) 273-8624
bbarbazuk@ufl.edu

Matias Kirst
2033 Mowry Road, room 320
Gainesville, FL, 32610
+1 (352) 846-0900
mkirst@ufl.edu

DOI: 10.1534/g3.113.008714

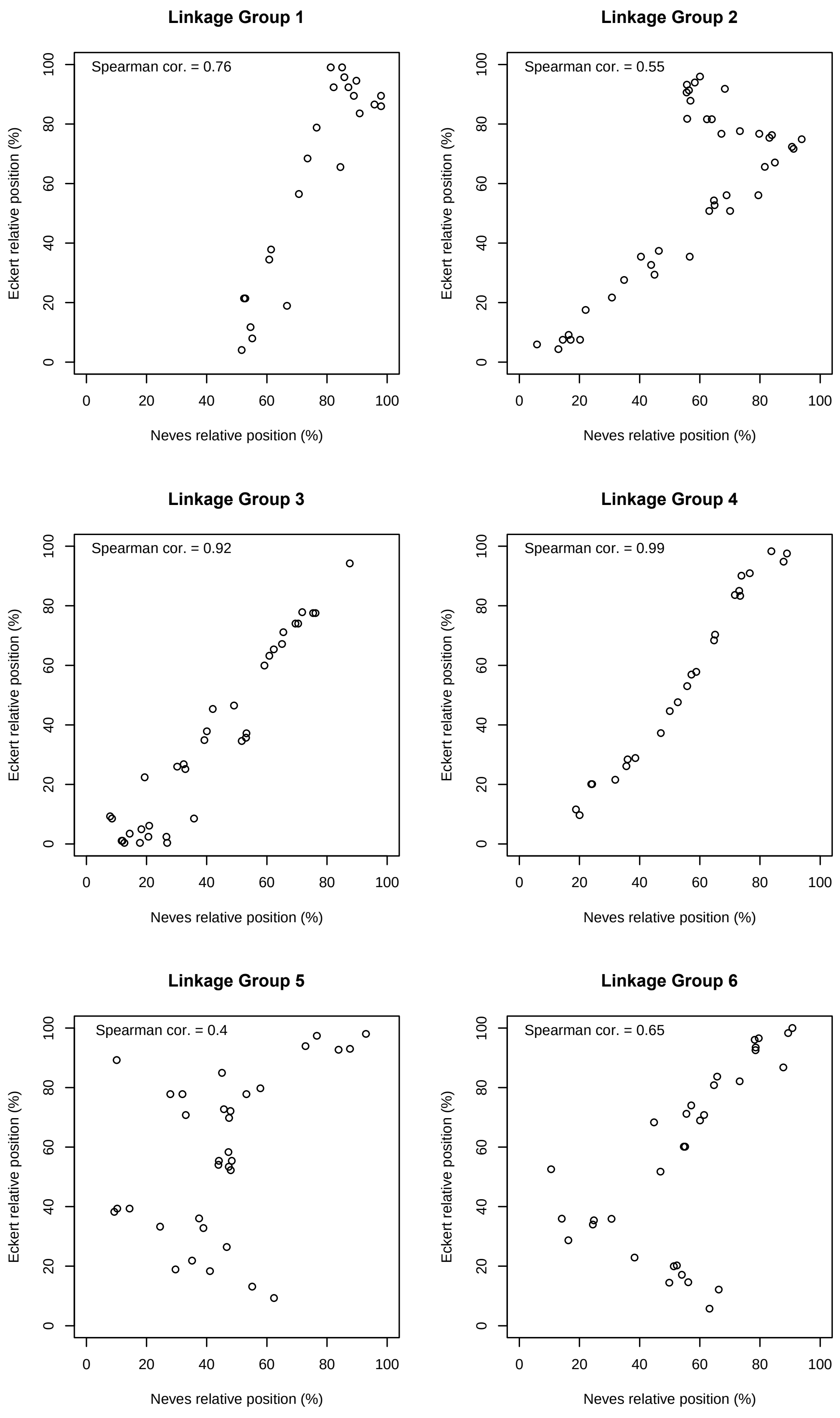

Figure S1 Comparison of the normalized relative order of shared genes used in our study (X-axis) and that of Eckert *et al.* (2009) (Y-axis) for linkage groups one to six. Assuming genes syntheny between the two populations, a straight line would illustrate perfect agreement at the gene ordering level between the two maps.

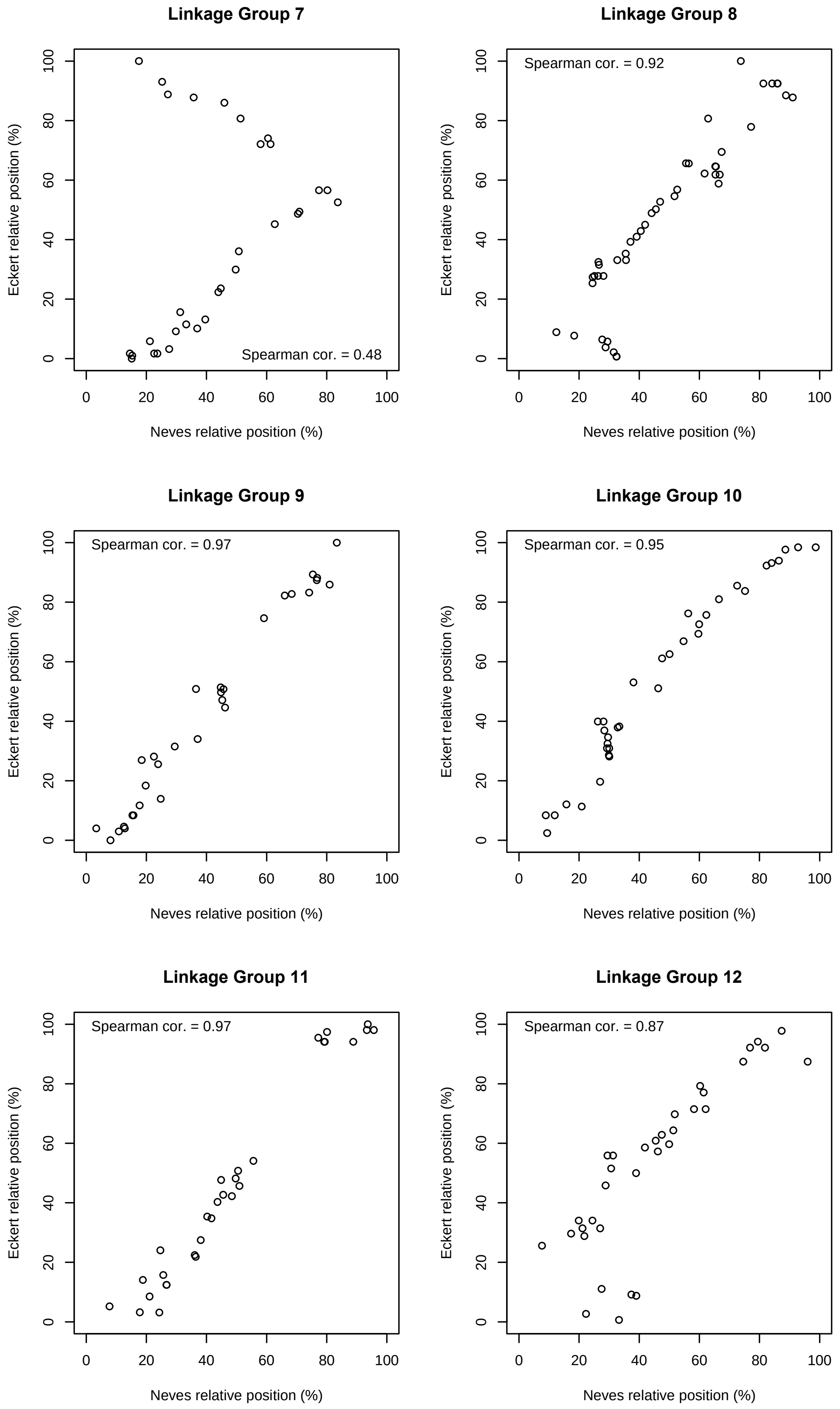

Figure S2 Comparison of the normalized relative order of shared genes used in our study (X-axis) and that of Eckert *et al.* (2009) (Y-axis) for linkage groups seven to twelve. Assuming genes syntheny between the two populations, a straight line would illustrate perfect agreement at the gene ordering level between the two maps.

Table S1 Sequencing strategy used for each multiplexed pool of eight haploid samples. Illumina's machine used and the respective cycle length used is shown.

Sample Pools	Titration run		Full runs		
	GAIIx 1x40bp	GAIIx 1x100bp	GAIIx 1x115bp	GAIIx 2x115bp	HiSeq 2x100bp
1	X			X	
2			X	X	
3			X	X	
4			X	X	
5			X	X	
6			X	X	
7			X	X	
8		X			
9					X

Tables S2-S4
Available for download as .csv files at http://www.g3journal.org/lookup/suppl/doi:10.1534/g3.113.008714/-/DC1

Table S2 Additional information about the 7,842 markers that segregated in the population. The last column describes the status of the marker in the mapping process, where "mapped_Rnd2" are markers that mapped at the second and third rounds of mapping; "mapped_Rnd3" are markers that only mapped at the third round of mapping; "not_tested_for_mapping" are markers for which another marker was selected to represent its gene for mapping and were not tested in the mapping step, and "ungrouped" are markers that did not pass the grouping step of JoinMap mapping.

Table S3 Text representation of the full genetic map with 2,841 genes mapped, containing the linkage group, the marker name and the position that marker mapped in the linkage group.

Table S4 Text representation of the genetic map of 1,371 genes mapped using the more conservative marker ordering of JoinMap Round 2. It contains the linkage group, the marker name and the position that marker mapped in the linkage group.
