# Supplementary material for: A High-Density Gene Map of Loblolly Pine (Pinus taeda L.) Based on Exome Sequence Capture Genotyping
Source: G3 (Bethesda). 2013 Nov 5;4(1):29–37. doi: 10.1534/g3.113.008714 (PMC3887537; doi:10.1534/g3.113.008714)
Supplement: Supporting Information [file supp_g3.113.008714_FigureS1.pdf]

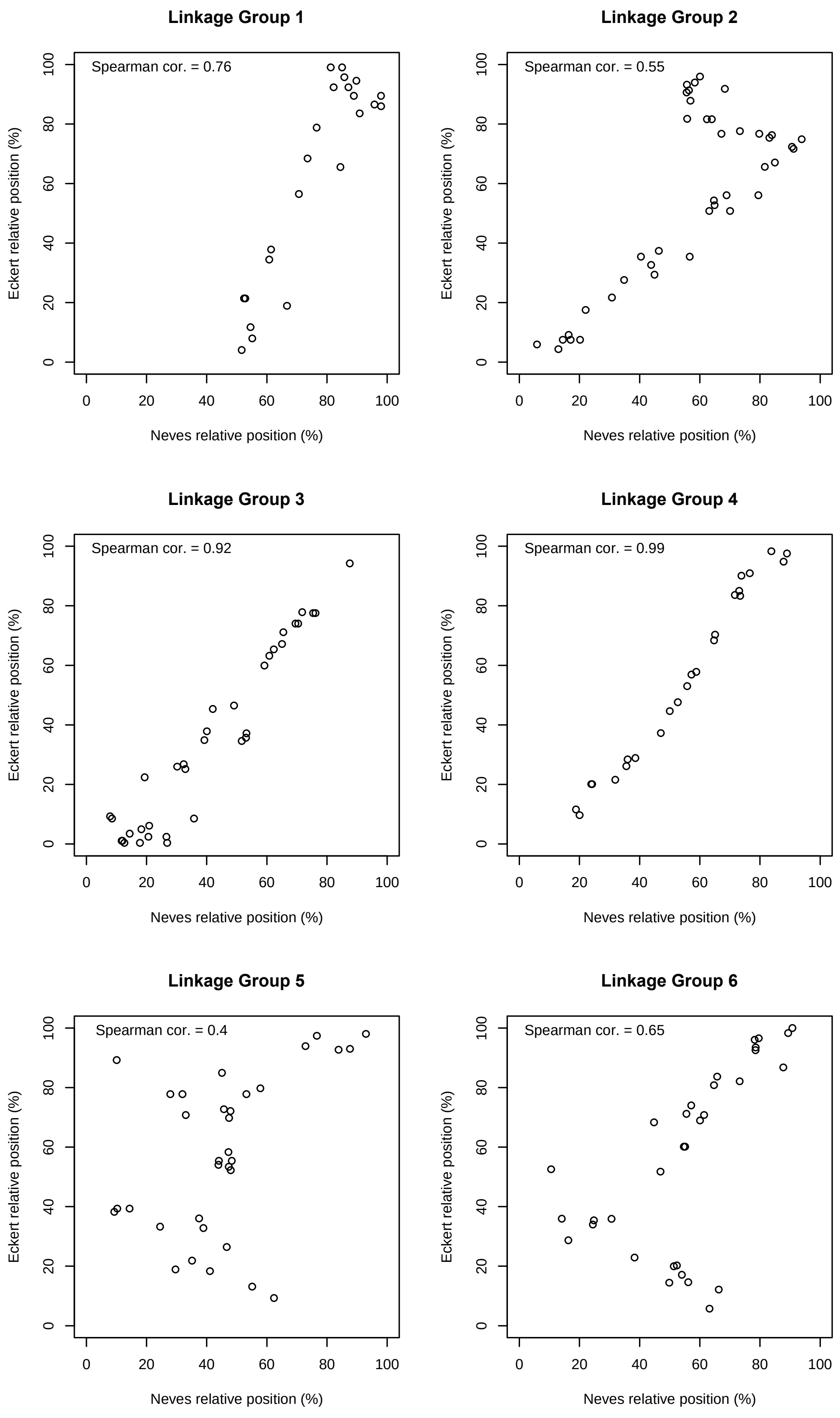

Figure S1 Comparison of the normalized relative order of shared genes used in our study (X-axis) and that of Eckert *et al.* (2009) (Y-axis) for linkage groups one to six. Assuming genes syntheny between the two populations, a straight line would illustrate perfect agreement at the gene ordering level between the two maps.
